# *Lactobacillus johnsonii* L531 Ameliorates *Salmonella enterica* Serovar Typhimurium Diarrhea by Modulating Iron Homeostasis and Oxidative Stress via the IRP2 Pathway

**DOI:** 10.3390/nu15051127

**Published:** 2023-02-23

**Authors:** Keyuan Chen, Jiufeng Wang, Liang Guo, Jing Wang, Lan Yang, Ting Hu, Yiqing Zhao, Xue Wang, Yaohong Zhu

**Affiliations:** 1 College of Veterinary Medicine, China Agricultural University, Beijing 100193, China; 2 Sanya Institute of China Agricultural University, Sanya 572000, China

**Keywords:** *Salmonella Typhimurium*, iron metabolism, *Lactobacillus johnsonii*, IRP2, oxidative stress

## Abstract

*Salmonella enterica* serovar Typhimurium (*S*. Typhimurium) has evolved mechanisms to evade the host’s nutritional immunity and thus promote bacterial growth by using the iron in the host. However, the detailed mechanisms of *S*. Typhimurium induce dysregulation of iron homeostasis and whether *Lactobacillus johnsonii* L531 can alleviate the iron metabolism disorder caused by *S*. Typhimurium has not been fully elucidated. Here, we show that *S*. Typhimurium activated the expression of iron regulatory protein 2 (IRP2), transferrin receptor 1, and divalent metal transporter protein 1 and suppressed the expression of iron exporter ferroportin, which resulted in iron overload and oxidative stress, inhibiting the key antioxidant proteins NF-E2-related factor 2, Heme Oxygenase-1, and Superoxide Dismutase in vitro and in vivo. *L. johnsonii* L531 pretreatment effectively reversed these phenomena. IRP2 knockdown inhibited iron overload and oxidative damage induced by *S*. Typhimurium in IPEC-J2 cells, while IRP2 overexpression promoted iron overload and oxidative damage caused by *S*. Typhimurium. Interestingly, the protective effect of *L. johnsonii* L531 on iron homeostasis and antioxidant function was blocked following IRP2 overexpression in Hela cells, demonstrating that *L. johnsonii* L531 attenuates disruption of iron homeostasis and consequent oxidative damage caused by *S*. Typhimurium via the IRP2 pathway, which contributes to the prevention of *S*. Typhimurium diarrhea in mice.

## 1. Introduction

As one of the zoonotic pathogens, *Salmonella enterica* serovar Typhimurium (*S*. Typhimurium) causes gastrointestinal diseases and systemic inflammatory reactions in humans and animals worldwide [1,2]. Among these, diarrhea in weaned piglets, one of the most typical clinical signs, has become a focus of attention in the field of health and safety of food of animal origin [3,4].

As a growth limiting factor, iron plays an essential role in the growth and reproduction of mammals and a wide range of pathogens [5]. As a highly iron-dependent pathogen, *Salmonella* uses iron from the host to promote its own intracellular survival and reproduction [6,7]. However, the detailed mechanisms of *S*. Typhimurium that induces dysregulation of host iron homeostasis have not been fully elucidated. Probiotics play an essential role in protecting the host from pathogens [8]. Recent studies have shown that *Lactobacillus* have a bi-directional effect on host iron homeostasis, and is even effective in relieving *S*. Typhimurium-induced intestinal damage in a high iron diet model [9]. *Lactobacillus johnsonii* L531, one of the *Lactobacillus*, has been demonstrated to be effective in alleviating *S*. Typhimurium-induced diarrhea, intestinal damage, and oxidative stress [3,10,11,12]. Nevertheless, it is unknown whether *L. johnsonii* L531 can reverse the iron metabolism disorder caused by *S*. Typhimurium.

During host iron homeostasis, the host acquires iron from the extracellular environment via the transferrin receptor (TfR1) and the divalent metal transporter protein (DMT1) [13], and transports free intracellular ferrous iron to the extracellular environment via iron exporter ferroportin (FPN, as the only mammalian protein that exports iron to the extracellular environment) to maintain a state of homeostasis in iron metabolism. The whole process is tightly controlled by the iron regulatory proteins IRP1 and IRP2, which act as central proteins regulating host iron homeostasis by binding to iron expression elements (IREs) located on DMT1, TfR1, and FPN to activate the expression of DMT and TfR1 and inhibit the expression of FPN [14]. Moreover, it is noted that high levels of free intracellular ferrous iron can lead to Fenton reaction inducing the production of reactive oxygen species (ROS), thereby causing oxidative stress [15,16]. Relevant studies have shown that *S*. Typhimurium causes the production of ROS and induces oxidative stress in the host [3,17]. However, it is unclear whether *S*. Typhimurium-induced oxidative stress is associated with iron metabolism disorder.

In response to oxidative stress, the host releases antioxidant factors to form a complex cascade of defensive signal responses, thereby ensuring cellular redox homeostasis [16]. Among these antioxidant factors, NF-E2-related factor 2 (Nrf2) plays a pivotal role in regulating the transcriptional expression of downstream antioxidant factors such as Heme Oxygenase-1 (HO-1), NAD(P)H: quinone oxidoreductase 1 (NQO1), Superoxide Dismutase (SOD), and Glutathione Peroxidase 1 (GPX1) [18,19,20]. Previous studies have reported that *L. johnsonii* L531 effectively alleviates oxidative stress caused by *Salmonella* [3]. However, whether this antioxidant function of *L. johnsonii* L531 is mediated through the iron metabolism pathway still needs to be further explored. 

Here, we used *S*. Typhimurium infection model in vitro and in vivo to evaluate the mechanisms by which *S*. Typhimurium induces disturbances in host iron metabolism and oxidative damage. Next, *L. johnsonii* L531 was used to reveal the exact protective effect on regulating iron homeostasis and *S*. Typhimurium-induced intestinal damage.

## 2. Materials and Methods

### 2.1. Ethics Statement

The animal study was reviewed and approved by the Guidelines for Laboratory Animal Use and Care from the Chinese Center for Disease Control and Prevention and the Rules for Medical Laboratory Animals (1998) from the Chinese Ministry of Health, under protocol AW32112202-2-1, approved by the Animal Ethics Committee of the China Agricultural University.

### 2.2. Chemicals and Antibodies

The following chemicals in this study were purchased from Solarbio (Beijing, China): phenylmethanesulfonyl fluoride (PMSF, P0100), Radio Immunoprecipitation Assay (RIPA) buffer (R0010), Protease Inhibitor Cocktail (539133), SDS-PAGE loading buffer (P1040), phosphate buffered solution with Tween 20 (PBST, P1033), Albumin Bovine (BSA, A8020), Triton X-100 (T8200), 4,6-diamidino-2-phenylindole (DAPI, C0065), 4% paraformaldehyde (PFA, P1110), and Mounting Medium, antifading (S2100). The following antibodies were used: Rabbit Anti-SLC40A1 antibody (bs-21360R, Bioss, Beijing, China), Anti-Transferrin Receptor antibody (ab214039, Abcam, Cambridge, UK), DMT1 Rabbit pAb (A10231, ABclonal, Wuhan, China), Anti-IREB2/IRP2 antibody (bs-4484R, Bioss, Beijing, China), Rabbit Anti-Aconitase 1 antibody (bs-9848R, Bioss, Beijing, China), Anti-Nrf2 antibody (ab137550, Abcam, Cambridge, UK), HO-1/HMOX1 Polyclonal antibody (10701-1-AP, Proteintech, Rosemont, IL, USA), and β-actin (66009-1-Ig). The secondary antibody HRP-labeled goat anti-rabbit IgG (H + L) (PR30009) and the HRP-labeled goat anti-mouse IgG (H + L) (HS201-01) were purchased from Proteintech (Rosemont, IL, USA). The Alexa Fluor 555-labeled secondary antibody (A0453) was purchased from Beyotime Biotechnology.

### 2.3. Bacterial Strains and Growth Conditions

The clinical isolates of *S*. Typhimurium used in this study were obtained from the feces or small intestinal contents from the diarrhea of piglets [21]. *L. johnsonii* L531 was isolated from the intestinal contents of healthy weaned piglets [22]. *S*. Typhimurium was transfected with the pFPV25.1 plasmids expressing green fluorescence to facilitate co-localization of *S*. Typhimurium with the cells. *S*. Typhimurium was grown in Luria–Bertani (LB) medium overnight at 37 °C with shaking at 200 rpm. On the next day, *S*. Typhimurium was inoculated in fresh LB broth (1% volume ratio) at 37 °C until the OD_600_ achieved 0.6 (logarithmic growth phase). *L. johnsonii* L531 was inoculated on De Man, Rogosa, and Sharpe (MRS, Land Bridge, Beijing, China) agar and incubated for 48 h at 37 °C.

### 2.4. Cell Culture

IPEC-J2 cells and Hela cells (stored in our laboratory) were grown in DME/F-12 1:1 (Cytiva, Marlborough, MA, USA) and supplemented with 10% fetal bovine serum and 1% penicillin/streptomycin (ThermoFish Scientific, Waltham, MA, USA) at 37 °C in a CO_2_ incubator with 5% CO_2_ and a humidified (95%) atmosphere.

### 2.5. S. Typhimurium Infection In Vitro

IPEC-J2 cells and Hela cells are widely used in in vitro models of *Salmonella* infection [3,6]. Before in vitro infection, IPEC-J2 cells and Hela cells were seeded at a density of 3 × 105 cells/well in 6-well culture plates and divided into 4 groups: (1) the control group (CN), (2) the *S*. Typhimurium group (ST, the multiplicity of infection (MOI) is 40), (3) the *L. johnsonii* L531 group (L, MOI, 10), and (4) the *L. johnsonii* L531+*S*. Typhimurium group (LS), *L. johnsonii* L531 for 3 h prior to the challenge of *S*. Typhimurium. After being treated with *S*. Typhimurium for 30 min, cells from all 4 groups were washed with PBS and supplemented with complete medium containing 100 μg/mL gentamicin for 2 h to kill the extracellular bacteria. Next, cells continued to be cultured in complete medium containing 10 μg/mL gentamicin to inhibit extracellular bacteria. 3-hydroxy-1,2-dimethyl-4(1H)-pyridone (deferiprone, DFP, an iron chelator for the treatment of iron overload [6], ID0980, Solarbio, Beijing, China) was used to treat the cells for 30 min before infection.

### 2.6. S. Typhimurium Infection In Vivo

A total of 40 6-week-old male C57BL/6 mice were obtained from Charles River Laboratory Animal Technology Co., Ltd. (Beijing, China). Mice were provided food and water ad libitum throughout the entire experiment. The experimental model and treatment regimens were appropriately modified according to the literature [3,23]. Briefly, similar to the experiment in vitro, the mice were also divided into 4 groups (*n* = 10 per group). For 7 days before *S*. Typhimurium challenge, mice in groups LS and L were orally inoculated with 5 × 10^8^ CFU of *L. johnsonii* L531 in 0.2 mL sterile physiologic saline, respectively; mice in groups CN and ST were administered an equal volume of sterile physiologic saline daily. On Day 8, the mice in groups ST and LS were given 0.2 mL of *S*. Typhimurium (3 × 10^6^ CFU per mice) by gavage, whereas mice in groups CN and L received 0.2 mL of sterile physiologic saline. The blood was harvested 3 days after infection, mice were then euthanized, and the ileum and liver tissue were collected to carry out histopathological examination. The feces and liver tissue were ground in sterile saline to quantify the bacterial load.

### 2.7. Assessment of Diarrhea Degree

The severity of diarrhea was evaluated using the dry/wet weight of fecal pellets. To determine the wet to dry weight ratio of feces, mice were placed in separate clean cages with no food or water provided. Next, 0.5 g of feces were collected and weighed. The feces were then placed in a 60 °C oven for 24 h until the change in weight was less than 1% and then weighed. The dry/wet weight ratio was then calculated.

### 2.8. Cell Viability Assay

The Cell Counting Kit-8 was used to determine IPEC-J2 cell viability. Briefly, IPEC-J2 cells were seeded in 96-well plates. An amount of 10 μL CCK-8 reagent was added to the cells at the appropriate time. Afterwards, the absorbance of the cell supernatant was measured at 450 nm using a UV spectrophotometer.

### 2.9. Enumeration of Intracellular Bacteria

To count the viable intracellular *S*. Typhimurium, cells were disrupted with 1% volume of Triton X-100. The serial dilutions of bacteria were plated on LB agar plates and incubated overnight at 37 °C.

### 2.10. Analysis of Intracellular S. Typhimurium Localization

IPEC-J2 cells were planted on cell slides and fixed in 4% paraformaldehyde for 10 min at 6 hpi, then the cells were treated with 1% Triton X-100 for 13 min. Next, DNA was stained with DAPI and then imaged with a Nikon A1 confocal laser scanning microscope.

### 2.11. Free Cellular Divalent Iron Content Assay

The cellular ferrous iron content (expressed as nmol of 10^6^ cells) was accessed by using the Ferrous Iron Colorimetric Assay Kit (E-BC-K773-M, Elabscience, Wuhan, China) in cells. Briefly, cells were collected and counted, followed by sonication to break up the cells to release the iron. The ferrous ions in the sample bind to the probe and the resulting substance has a strong absorption peak at 593 nm, and its optical density value is linearly correlated with the concentration of ferrous iron over a range of wavelengths.

### 2.12. Iron Measurement in the Serum and Tissue

Serum iron (expressed as μg/dL of serum) and tissue iron content (expressed as μmol/g of protein) were measured with a Micro Serum Iron Concentration Assay Kit (BC1735, Solarbio, Beijing, China) and a Tissue Iron Assay Kit (A039-2-1, Nanjing Jiancheng Bioengineering Institute, Nanjing, China) according to the manufacturer’s instructions, respectively.

### 2.13. Assessment of the Antioxidant Status in Tissue

SOD (expressed as units/mg of protein), Total Antioxidant Capacity (T-AOC) (expressed as mmol/g of protein), and MDA (expressed as nmol/mg of protein) were measured in liver and ileum using commercial kits (S0101S and S0121, Beyotime, Beijing, China; A003-1-2, Nanjing Jiancheng Bioengineering Institute, Nanjing, China). The SOD, T-AOC activity, and MDA level were detected following the manufacturer’s instructions.

### 2.14. Iron Staining

The IPEC-J2 cells or Hela cells planted on plates were incubated with 1 μmol/L FerroOrange (Dojindo, F374, Shanghai, China) working solution (prepared with DME/F-12 1:1) for 30 min, and then imaged with a Nikon A1 confocal laser scanning microscope.

### 2.15. Assessment of Intracellular ROS Generation

The cells were washed 3 times with DME/F-12 1:1 at 6 hpi. The working solution DCFH-DA (10 μM) was then added to IPEC-J2 cells for 30 min. Imaging was performed with a Nikon Eclipse Ti-U fluorescent microscope.

### 2.16. Dihydroethidium Staining

Dihydroethidium assay kit (Beyotime, Shanghai, China) was used to detect the level of ROS in Hela cells. The working solution Dihydroethidium (5 μM) was added to the Hela cells for 30 min after the treatments. Imaging was performed with a Nikon Eclipse Ti-U fluorescent microscope.

### 2.17. siRNA and Transfection Experiments

The negative control siRNA (siCN, sense 5′-UUCUCCGAACGUGUCACGUTT-3′; antisense 5′-ACGUGACACGUUCGGAGAATT-3′) and siRNA specific for IRP2 (siIRP2, sense 5′-GCAAUACAGAAUGCUCCAATT-3′; antisense 5′-UUGGAGCAUUCUGUAUUGCTT-3′) were purchased from GenePharma (Shanghai, China). IPEC-J2 cells were transfected with siRNAs using Lipofectamine RNAiMAX reagent (13778150, Thermo Fisher Scientific, Waltham, MA, USA) for 48 h before the *S*. Typhimurium challenge.

In plasmid transfection experiments, pEGFP-C1 vectors were stored in our laboratory. To obtain the recombinant plasmid pEGFP-C1-IRP2, IRP2 gene was ligated to pEGFP-C1 with specific primers (Table 1). The plasmids were transfected with Lipofectamine™ 3000 Transfection Reagent (L3000015, Thermo Fisher Scientific) according to the manufacturer’s instructions. *S*. Typhimurium challenge was performed after 36 h post-transfection.

### 2.18. Western Blotting

Tissue and cells were lysed using RIPA buffer containing a 1% protease/phosphatase inhibitor cocktail (Cell Signaling Technology, Danvers, MA, USA). Protein concentrations were determined with a BCA kit. Protein extracts were separated using SDS-PAGE and then transferred to polyvinylidene fluoride membranes. After being blocked with 5% BSA, the membranes were then incubated with primary antibodies at 4 ℃ overnight: IRP2 (1:1000), IRP1 (1:1000), DMT1 (1:1000), TfR1 (1:1000), FPN (1:1000), Nrf2 (1:1000), and HO-1 (1:5000). The blots were then incubated with the horseradish peroxidase-labelled secondary antibodies (1:5000) for 45 min at room temperature. The blots were coated with ECL immunoblotting substrate for development and images were captured using a Tanon 6200 chemiluminescence imaging workstation.

### 2.19. Immunofluorescence

Ileal and hepatic tissues were fixed in 4% formaldehyde overnight and were then embedded and implanted into paraffin blocks. IPEC-J2 cells were planted on glass coverslips in culture plates. Cells were fixed with 4% paraformaldehyde for 10 min. Next, tissues and cells were washed with PBS followed by incubation with 1% Triton X-100 for 13 min, then were blocked with 2% BSA for 1 h at room temperature for reducing non-specific background. Tissues and cells were then incubated with anti-IRP2 antibody at 4 °C overnight. Finally, the tissues and cells were treated with the appropriate Alexa Fluor 555-labeled secondary antibody for 45 min and then incubated with DAPI for 3 min at room temperature. Photomicrographs were taken with a Nikon A1 confocal laser scanning microscope.

### 2.20. RNA Extraction and qRT-PCR

Total RNA was isolated from IPEC-J2 cells and Hela cells using RNAiso Plus (Takara, SanJose, CA, USA). To obtain cDNA, reverse transcription reactions were performed using the PrimeScriptTM RT kit (RR047A, TaKaRa, Kyoto, Japan) following the manufacturer’s instructions. Primer sequences for PCR are listed in Table 1. Circulating thresholds (CT) values of target genes for CT were treated with CT values for hypoxanthine phosphoribosyl-transferase housekeeping according to the normalization principle. The results were indicated in fold-change by using the 2^−ΔΔCT^ method.

### 2.21. Statistical Analysis

IBM SPSS statistics 23.0 (Chicago, IL, USA) was used for statistical analysis. Data are presented as mean ± SEM. Comparisons between the two groups were made using the independent samples *t*-test, and one-way ANOVA was used to analyze data from multiple two groups followed by Tukey’s multiple comparison test. *p* < 0.05 was considered statistically significant.

## 3. Results

### 3.1. L. johnsonii L531 Reverses the Disruption of Iron Metabolism Caused by S. Typhimurium in IPEC-J2 Cells

**Figure 1:**
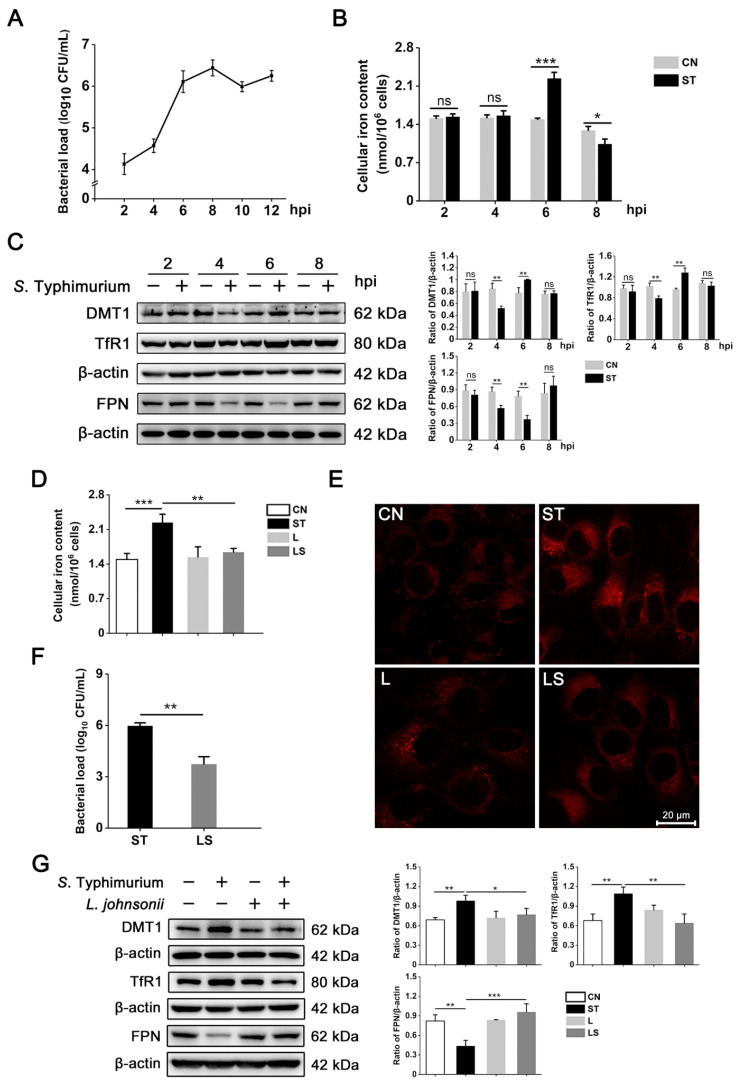
*L. johnsonii* L531 reverses the disruption of iron metabolism caused by *S*. Typhimurium in IPEC-J2 cells. (**A**) *S*. Typhimurium (MOI = 40) burden was tested by plating. Data were obtained from three independent replicates of each sample at 2, 4, 6, 8, 10, and 12 hpi. hpi: hours post infection. (**B**,**D**) The cellular free iron content of IPEC-J2 cells. CN, Control. ST, *S*. Typhimurium. L, *L. johnsonii* L531. LS, *L. johnsonii* L531 + *S*. Typhimurium. (**C**,**G**) Western blot analysis of DMT1, TfR1, and FPN. (**E**) Free ferrous iron in IPEC-J2 cells was stained by FerroOrange. Scale bar, 20 μm. (**F**) The bacterial load was detected by plating. Values are expressed as the means ± SEM of 3 independent experiments. * *p* < 0.05, ** *p* < 0.01, *** *p* < 0.001 (ns, not significant).

We firstly used the IPEC-J2 cell model to investigate the proliferation of *S*. Typhimurium. The curve showed that *S. Typhimurium* began to rapidly multiply at 4 h post infection (hpi), and the amplitude of proliferation decreased at 8 hpi (Figure 1A). Therefore, we focused on the 0–8 hpi that encompassed the peak of bacterial replication and measured the intracellular free ferrous iron content during this period. Compared with uninfected cells, *S*. Typhimurium significantly increased the content of iron in IPEC-J2 cells at 6 hpi but did not cause intracellular iron accumulation at 4 hpi and 8 hpi, respectively (Figure 1B).

To gain further insights into the effect of *S*. Typhimurium infection on iron metabolism in cells, we detected the expression of three key iron transport proteins by western blot. Compared with the control group, the expression of DMT1 and TfR1 was significantly upregulated at 6 hpi, and FPN was obviously suppressed at the same time point of infection (Figure 1C). However, *L. johnsonii* L531 pretreatment effectively attenuated *S*. Typhimurium-induced iron accumulation and reduced intracellular bacterial load (Figure 1D–F). Additionally, *L. johnsonii* L531 pretreatment obviously suppressed the upregulation of DMT1 and TfR1 and reversed the downregulation of FPN caused by *S*. Typhimurium (Figure 1G,H). Altogether, these data clearly indicate that *L. johnsonii* L531 are effective in alleviating iron metabolism disorders induced by *S*. Typhimurium in IPEC-J2 cells.

### 3.2. S. Typhimurium Promotes Its Growth and Triggers Oxidative Stress by Inducing the Disturbance of Iron Homeostasis in IPEC-J2 Cells

**Figure 2:**
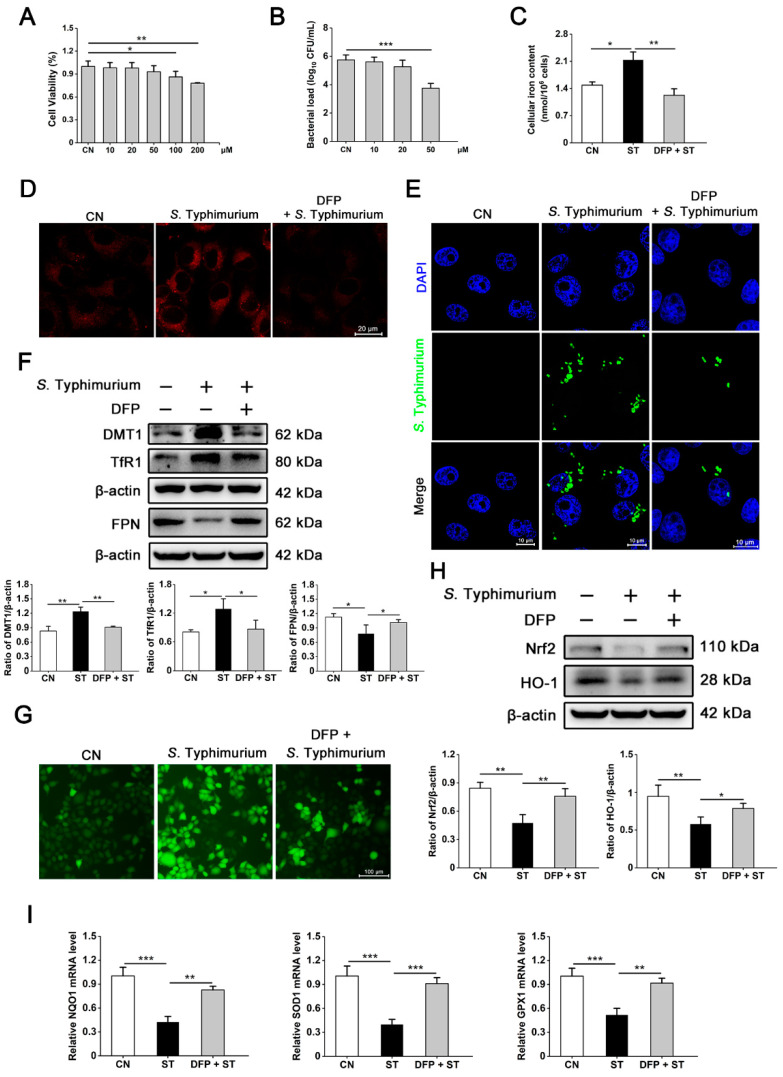
Iron homeostasis disorder triggered by *S*. Typhimurium promotes its growth and provokes oxidative stress in IPEC-J2 cells. (**A**) Cell viability assay of IPEC-J2 cells. CN, Control. (**B**) The intracellular bacterial load in IPEC-J2 Cells. (**C**) The cellular free iron content of IPEC-J2 cells. (**D**) Free ferrous iron fluorescence staining. Scale bars, 20 μm. (**E**) Analysis of *S*. Typhimurium intracellular localization. Blue, DAPI. Green, *S*. Typhimurium. Scale bars, 10 μm. Western blot analysis of DMT1, TfR1, FPN (**F**), Nrf2, and HO-1 (**H**). (**G**) ROS levels were determined by DCFH-DA kit. Scale bars, 100 μm. (**I**) The NQO1, SOD1, and GPX1 mRNA expression levels in IPEC-J2 cells. Values are expressed as the means ± SEM of 3 independent experiments. * *p* < 0.05, ** *p* < 0.01, *** *p* < 0.001.

To further study the regulatory role of iron homeostasis during *S*. Typhimurium infection, we used the iron chelator DFP to treat cells prior to infection. Firstly, we screened the optimum concentration of DFP for treatment, and found that the addition of 50 μM DFP not only did not affect cell viability but also significantly inhibited the intracellular load of *S*. Typhimurium (Figure 2A,B). Thus, 50 μM DFP was used for the subsequent experiments. Pretreatment with DFP significantly alleviated the accumulation of iron caused by *S*. Typhimurium, which was further corroborated by cellular free divalent iron staining (Figure 2C,D). Intracellular localization analysis of *S*. Typhimurium likewise showed an inhibitory effect of DFP on bacterial growth (Figure 2E). Next, the differences in expression abundance of iron homeostasis-related proteins were detected. DFP pretreatment efficiently reversed the abnormal upregulation of DMT1 and TfR1 and downregulation of FPN protein caused by *S*. Typhimurium infection (Figure 2F). These findings indicate that *S*. Typhimurium promotes its own replication by inducing dysregulation of iron metabolism in IPEC-J2 cells.

Additionally, we also observed that *S*. Typhimurium triggered the accumulation of intracellular ROS compared to the control group, which could also be alleviated by DFP (Figure 2G). Among them, we next performed the expression levels of antioxidant protein Nrf2 and its downstream target protein HO-1 and the mRNA expression levels of target genes NQO1, SOD1, and GPX1. Interestingly, DFP pretreatment effectively alleviated the inhibition of expression of these key antioxidant proteins and genes caused by *S*. Typhimurium infection (Figure 2H,I). Taken together, these results suggest that dysregulation of cellular iron metabolism triggered by *S*. Typhimurium further contributes to oxidative stress.

### 3.3. IRP2, but Not IRP1, Is Involved in Regulating the Dysregulation of Iron Metabolism Induced by S. Typhimurium in IPEC-J2 Cells

**Figure 3:**
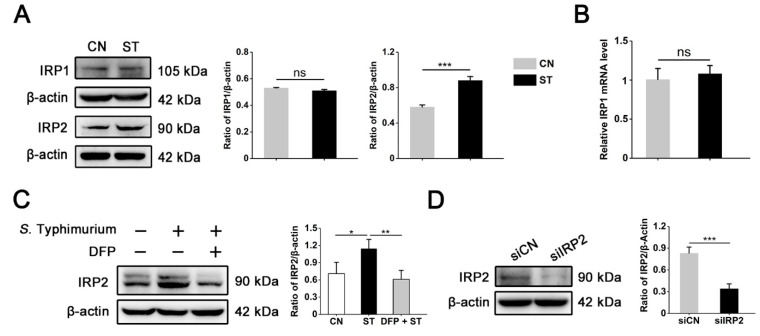

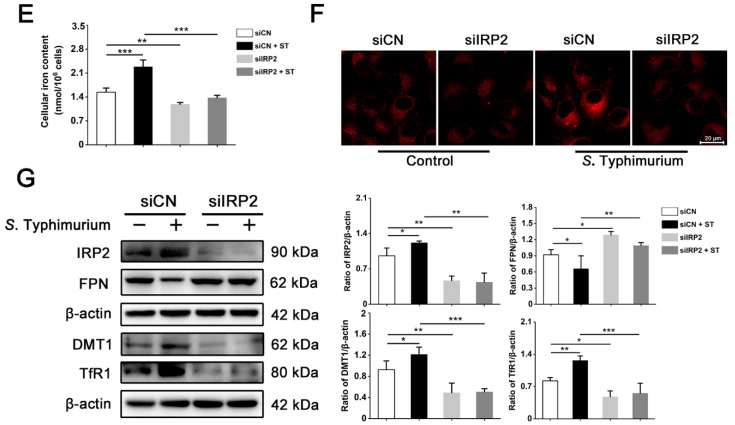
IRP2, but not IRP1, is involved in regulating the dysregulation of iron metabolism induced by *S*. Typhimurium in IPEC-J2 cells. (**A**) Western blot analysis of IRP1 and IRP2 after the *S*. Typhimurium challenge. (**B**) The IRP1 mRNA expression levels. (**C**) Western blot analysis of IRP2. Cells were treated with DFP for 30 min before *S*. Typhimurium infection. (**D**) Western blot analysis of IRP2 at 48 h post-transfection. siCN, siControl. (**E**) Detection of the cellular free iron content. Cells were transfected with siRNAs for 48 h before the *S*. Typhimurium challenge. (**F**) Free ferrous iron fluorescence staining to assess iron content. Scale bar, 20 µm. (**G**) Western blot analysis of IRP2, DMT1, and TfR1. Values are expressed as the means ± SEM of 3 independent experiments. * *p* < 0.05, ** *p* < 0.01, *** *p* < 0.001 (ns, not significant).

To further determine whether *S*. Typhimurium regulates host cell iron homeostasis through the IRPs system, we next detected the expression levels of IRP1 and IRP2 at 6 hpi, respectively. Compared with the control group, the expression abundance of IRP2 was drastically increased in the *S*. Typhimurium-infected group, while the protein and mRNA expression levels of IRP1 did not show obvious differences (Figure 3A,B). More importantly, DFP pretreatment reversed the *S*. Typhimurium-induced activation of IRP2 (Figure 3C), suggesting that IRP2 was involved in regulating *S*. Typhimurium-induced dysregulation of iron metabolism. Among these, in order to clarify the role of IRP2 in iron metabolism during *S*. Typhimurium infection, we depleted the levels of IRP2 in cells by siRNA. Western blot analysis showed that siRNA effectively inhibited the expression of IRP2 (Figure 3D). Subsequently, we observed that knockdown of IRP2 further reduced the cellular iron accumulation caused by *S*. Typhimurium (Figure 3E,F). We next explore the effect of IRP2 knockdown on expression abundance of iron transporter protein by western blot during infection. Intriguingly, knockdown of IRP2 also decreased the expression of DMT and TfR1 and increased the abundance of FPN. More notably, compared to the infected control group (siCN + ST), knockdown of IRP2 remarkably blocked the effect of activation on DMT1, TfR1, and IRP2, and inhibition on FPN caused by *S*. Typhimurium (Figure 3G). Together, our results uncover a mechanism by which *S*. Typhimurium induces disruption of iron metabolism via the IRP2 pathway.

### 3.4. S. Typhimurium Promotes Its Growth and Induces Oxidative Stress via the IRP2 Pathway in IPEC-J2 Cells

**Figure 4:**
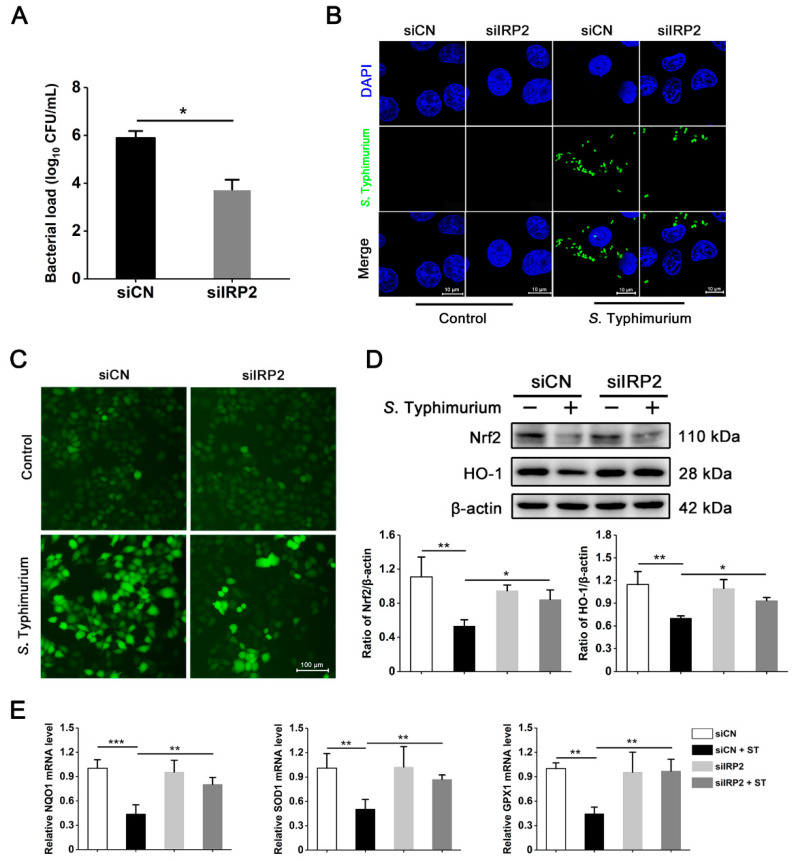
*S*. Typhimurium promotes its growth and induces oxidative stress via the IRP2 pathway in IPEC-J2 cells. (**A**) The intracellular bacterial load. (**B**) Analysis of *S*. Typhimurium intracellular localization. Blue, DAPI. Green, *S*. Typhimurium. Scale bar, 10 μm. (**C**) ROS levels. Scale bar, 100 μm. (**D**) Western blot analysis of Nrf2 and HO-1. (**E**) The detection of mRNA expression levels on NQO1, SOD1, and GPX1. Values are expressed as the means ± SEM of 3 independent experiments. * *p* < 0.05, ** *p* < 0.01, *** *p* < 0.001.

We have observed that *S*. *typhimurium* could promote its intracellular replication and induce oxidative stress by perturbing disturbed iron homeostasis (Figure 2). Thus, to further investigate the regulatory role played by IRP2 in this process, we next used knockdown of IRP2 to determine bacterial count and localization of *S*. Typhimurium. As shown in Figure 4A,B, knockdown of IRP2 significantly reduced the intracellular load of *S*. Typhimurium. Further, in the *S*. Typhimurium challenge groups, interfering with the expression of IRP2 could effectively relieve ROS production and inhibition on antioxidant proteins Nrf2 and HO-1 (Figure 4C,D); the mRNA expression levels of antioxidants NQO1, SOD1, and GPX1 showed a similar trend to Nrf2 (Figure 4E). These analyses reveal that *S*. Typhimurium facilitates its own intracellular reproduction and triggers the onset of oxidative stress through the IRP2 pathway in IPEC-J2 cells.

### 3.5. L. johnsonii L531 Suppresses the High Expression of IRP2 and Alleviates Oxidative Stress Caused by S. Typhimurium

**Figure 5:**
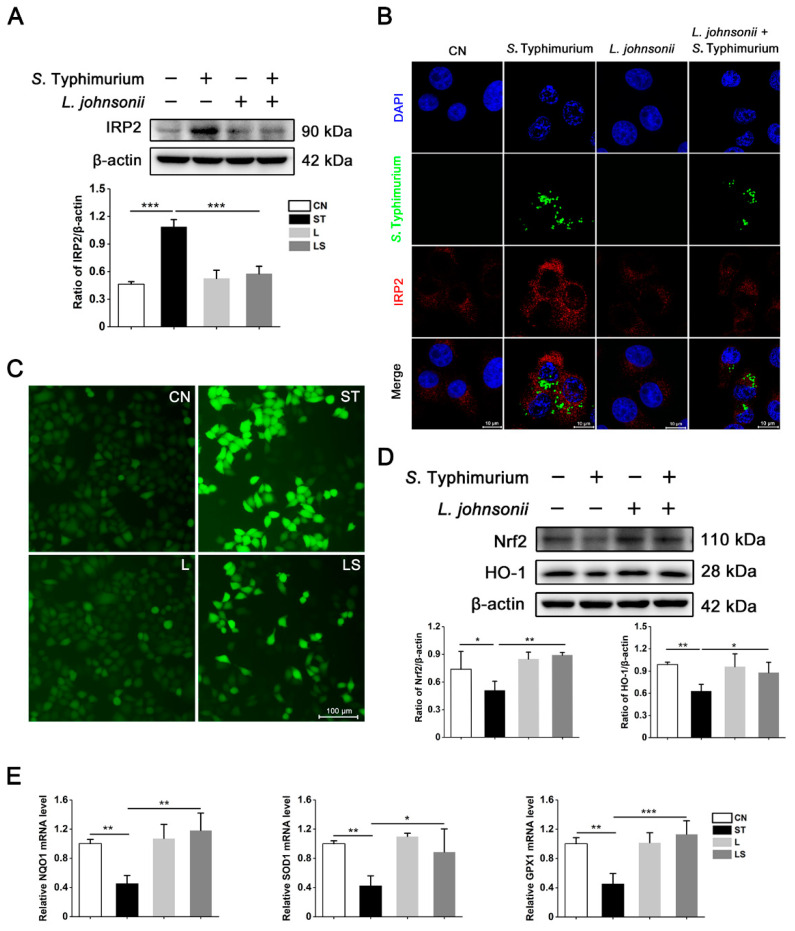
*L. johnsonii* L531 suppresses the high expression of IRP2 and alleviates oxidative stress caused by *S*. Typhimurium. (**A**) Western blot analysis of IRP2. (**B**) Distribution of IRP2 (Red) and *S*. Typhimurium (Green) in IPEC-J2 cells by immunofluorescence staining. Blue: DAPI. Scale bar, 10 μm. (**C**) DCFH-DA staining to assess ROS. Scale bar, 100 μm. (**D**) Western blot analysis of Nrf2 and HO-1. (**E**) The NQO1, SOD1, and GPX1 mRNA expression levels. Values are expressed as the means ± SEM of 3 independent experiments. * *p* < 0.05, ** *p* < 0.01, *** *p* < 0.001.

We have validated that *L. johnsonii* L531 could dramatically alleviate *S*. Typhimurium-induced iron metabolism disorders based on the pivotal role of IRP2 in *S*. Typhimurium-induced iron metabolism disorders and oxidative stress. Therefore, we further explored the effect of *L. johnsonii* L531 on IRP2 protein expression and oxidative stress. As shown in Figure 5A, *L. johnsonii* L531 pre-incubation significantly suppressed the upregulation of IRP2 caused by *S*. Typhimurium. As further confirmation, it is more intuitive that *S*. Typhimurium obviously increased the expression of IRP2 and finished its own replication, while pretreatment with *L. johnsonii* L531 effectively reversed this phenomenon (Figure 5B). Moreover, *L. johnsonii* L531 pretreatment also reduced the level of ROS caused by *S*. Typhimurium (Figure 5C) and upregulated the expression levels of Nrf2 and HO-1 compared to the *S*. Typhimurium challenge groups (Figure 5D). Meanwhile, the mRNA expression levels of NQO1, SOD1, and GPX1 also showed a similar trend to Nrf2 and HO-1 (Figure 5E). Collectively, these data clearly suggest that *L. johnsonii* L531 has a crucial role in suppressing the activation of IRP2 and mitigating oxidative stress caused by *S*. Typhimurium in IPEC-J2 cells, and the results imply that *L. johnsonii* L531 exerts it’s protective effect through the IRP2 pathway.

### 3.6. L. johnsonii L531 Relieves Dysregulation of Iron Homeostasis and Oxidative Stress Caused by S. Typhimurium via IRP2 in Hela Cells

**Figure 6:**
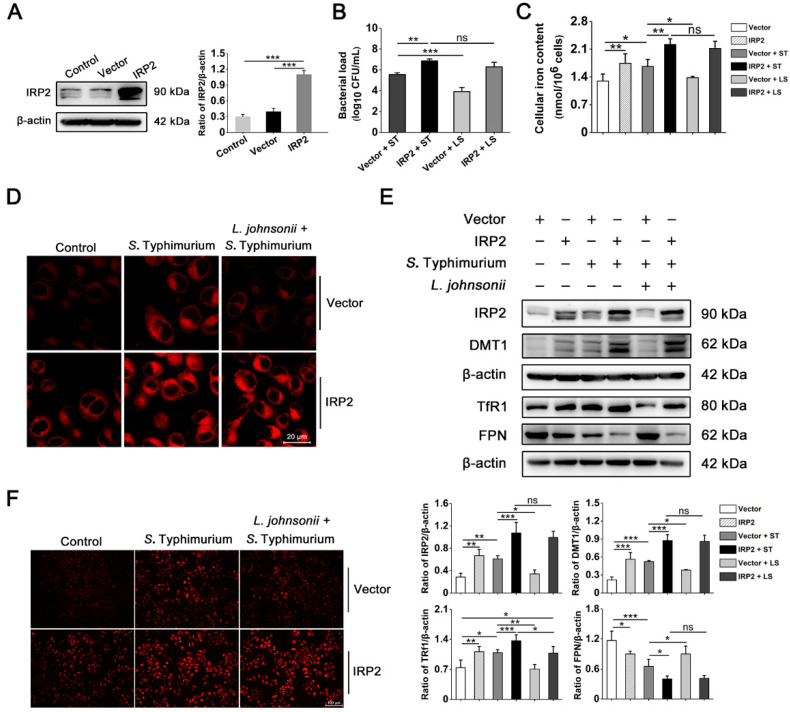

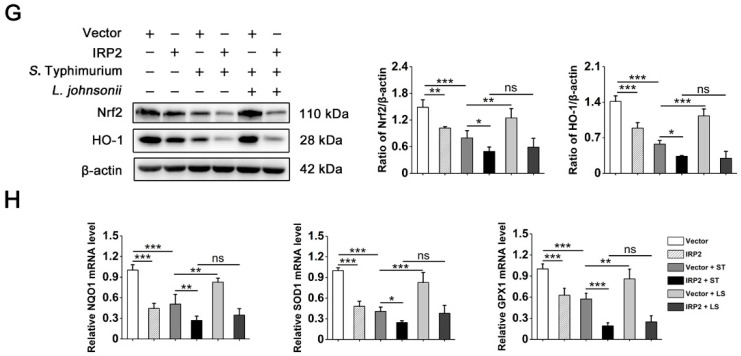
*L. johnsonii* L531 relieves dysregulation of iron homeostasis and oxidative stress caused by *S*. Typhimurium via IRP2 in Hela cells. (**A**) Western blot analysis of IRP2. (**B**) The intracellular bacterial count was tested by plating. (**C**) The cellular free iron content. (**D**) Free ferrous iron fluorescence staining. Scale bar, 20 μm. (**E**) Western blot analysis of IRP2, DMT1, TfR1, and FPN. (**F**) DHE staining to assess ROS level. Scale bar, 100 μm. (**G**) Western blot analysis of Nrf2 and HO-1. (**H**) The NQO1, SOD1, and GPX1 mRNA expression levels. Values are expressed as the means ± SEM of 3 independent experiments. * *p* < 0.05, ** *p* < 0.01, *** *p* < 0.001 (ns, not significant).

To further determine whether *L. johnsonii* L531 exerts protective effect via the IRP2 pathway, a vector allowing strong and constitutive expression of the IRP2 gene was constructed and transformed into HeLa cells for 36 h before *S*. Typhimurium challenge or *L. johnsonii* L531 pretreatment. As shown in Figure 6A, the protein abundance of the IRP2 group (IRP2) was significantly increased compared with the control group and the empty vector group (Vector), indicating that the IRP2 eukaryotic expression vector was successfully constructed. Further, we investigated the effect of overexpression of IRP2 on the process of *S*. Typhimurium infection. Intriguingly, *L. johnsonii* L531 could effectively inhibit *S*. Typhimurium-induced cellular iron accumulation and intracellular replication, while this protective effect seemed to disappear after overexpression of IRP2 (Figure 6B–D). In addition, cells overexpressing IRP2 exhibited a similar phenotype to the *S*. Typhimurium-infected group (Vector + ST) compared with cells transfected with the empty vector and compared with the *S*. Typhimurium-infected group, overexpression of IRP2 followed by infection resulted in a further increase in iron content and bacterial load (Figure 6B–D). Moreover, western blot analysis of iron transport protein showed that both *S*. Typhimurium and overexpression of IRP2 promoted the expression of DMT1 and TfR1 and suppressed the expression of FPN, which was similarly exacerbated by overexpression followed by *S*. Typhimurium infection. Similar to the above results, although pre-incubation with *L. johnsonii* L531 could reverse the abnormal expression of iron transporter proteins caused by *S*. Typhimurium, it did not have the efficient protective effect on *S*. Typhimurium infection after overexpression of IRP2 (Figure 6E). These data further illustrate the critical role of IRP2 in promoting the intracellular replication of *S*. Typhimurium, remarkably, and clearly reveals the special protective effect of *L. johnsonii* L531 on alleviating *S*. Typhimurium-induced disorders of iron metabolism via the IRP2 pathway.

Subsequently, to confirm whether *L. johnsonii* L531 mitigates oxidative stress via the IRP2 pathway, we next performed a DHE staining to access the ROS production. Similar to the cellular iron content results described in Figure 6C, both IRP2 overexpression and *S*. Typhimurium infection resulted in high ROS, and *L. johnsonii* L531 pretreatment effectively reversed this stress response induced by *S*. Typhimurium. Furthermore, the stress response induced by *S*. Typhimurium infection following IRP2 overexpression was similarly further enhanced. In contrast, *L. johnsonii* L531 cannot exert a substantial protective effect at this time (Figure 6F). Additionally, as further confirmation, the expression levels of the antioxidant proteins Nrf2 and HO-1 and genes NQO1, SOD1, and GPX1 showed a similar trend to DHE staining. Protein and mRNA expression analysis of the antioxidant protein and genes revealed that although *L. johnsonii* L531 attenuated the decrease in expression levels induced by *S*. Typhimurium infection in cells transfected with the empty vector, this protective effect eliminated after IRP2 overexpression. Meanwhile, overexpression of IRP2 also inhibited the expression to some extent and this effect was exacerbated by infection with *S*. Typhimurium after overexpression of IRP2 (Figure 6G,H). Taken together, our findings further confirm that *S*. Typhimurium disrupts the cellular antioxidant system through IRP2 and triggers oxidative stress. More particularly, these data demonstrate that *L. johnsonii* L531 plays an antioxidant role through the IRP2 pathway.

### 3.7. L. johnsonii L531 Ameliorates Disturbance of Iron Metabolism and Diarrhea Induced by S. Typhimurium In Vivo

**Figure 7:**
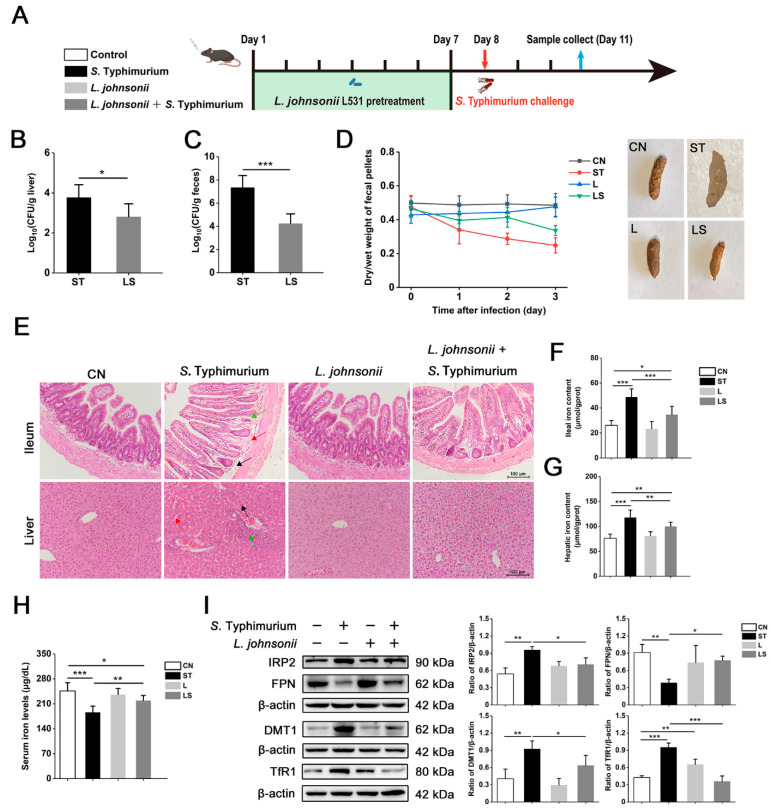

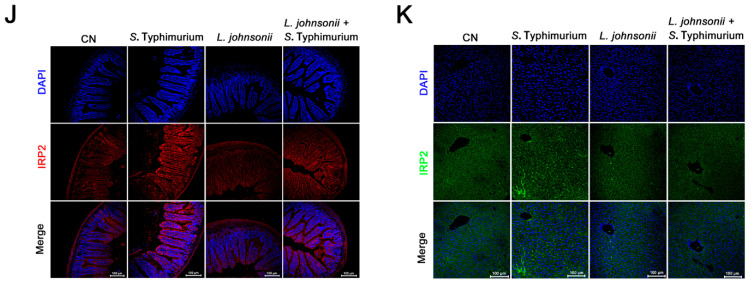
*L. johnsonii* L531 ameliorates disturbance of iron metabolism and diarrhea induced by *S*. Typhimurium in vivo. (**A**) Experimental design. Viable cell count of intracellular *S*. Typhimurium in liver (**B**) and feces (**C**) was determined by plating (*n* = 6 mice, respectively). (**D**) The results of the dry/wet weight ratio of the feces. (**E**) Ileum and liver representative photomicrographs of sections stained with H&E. In the ileum, the green arrow indicates atrophy of ileal intestinal glands, red arrow indicates basement membrane mucosal oedema, and black arrow indicates intestinal glands detached from basement membrane. In the liver, the red arrow indicates disturbed hepatocyte arrangement, green arrow indicates inflammatory cell infiltration, and black arrow indicates hepatocyte degeneration and necrosis. Ileum (**F**), hepatic (**G**), and serum iron (**H**) levels (*n* = 6 mice, respectively). (**I**) Western blot analysis of IRP2, DMT1, TfR1, and FPN in ileum. Distribution of IRP2 in ileum (**J**) and liver (**K**) by immunofluorescence staining. IRP2: Red (Ileum), Green (Liver), Blue: DAPI. Scale bar, 100 μm. Values are expressed as the means ± SEM of 3 independent experiments. * *p* < 0.05, ** *p* < 0.01, *** *p* < 0.001.

To assess the preventive effect of *L. johnsonii* L531 on *S*. Typhimurium infection in vivo, we established a mouse model of diarrhea caused by *S*. Typhimurium infection (Figure 7A). Our results showed that *L. johnsonii* L531 pretreatment obviously reduced *S*. Typhimurium burden in the liver and feces (Figure 7B,C). Concurrently, compared to the control group, *S*. Typhimurium-infected mice had soft and irregular stools with a significantly lower wet-to-dry weight ratio, which indicated that *S*. Typhimurium caused the mice to develop diarrhea (Figure 7D). We next performed a pathological analysis of the ileum and liver, as shown in Figure 7E. The *S*. Typhimurium-infected group showed atrophy of ileal intestinal glands, intestinal glands detached from basement membrane, and basement membrane mucosal oedema, whereas *L. johnsonii* L531 pretreatment could effectively alleviate these pathological changes. Further, *L. johnsonii* L531 pretreatment could also significantly reverse inflammatory cell infiltration, disturbed hepatocyte arrangement, hepatocyte degeneration, and necrosis caused by *S*. Typhimurium in the liver. In addition, *S*. Typhimurium caused an increase of iron content in the ileal and liver, and a decrease in serum iron content, indicating that *S*. Typhimurium infection induced a disturbance in iron metabolism in mice. Interestingly, *L. johnsonii* L531 was effective in alleviating this phenomenon, and although *L. johnsonii* L531 failed to restore iron levels to those of the control group, it was significantly improved compared with the *S*. Typhimurium group. Among these findings, we further detected the expression levels of iron transporter proteins. In line with our in vitro observations (Figure 1G and Figure 5A), the protein expression levels of IRP2, DMT1, and TfR1 were significantly activated and FPN was markedly inhibited in the ileum and liver, however, *L. johnsonii* L531 could mitigate relatively normal expression levels of these key proteins (Figure 7I and Figure S1A). Immunofluorescence analysis further showed the inhibitory effect of *L. johnsonii* L531 on the high expression of IRP2 caused by *S*. Typhimurium in the ileum and liver (Figure 7J,K). Together with these results, *L. johnsonii* L531 plays a beneficial role in ameliorating disruption of iron metabolism and diarrhea induced by *S*. Typhimurium in vivo.

### 3.8. L. johnsonii L531 Relieves S. Typhimurium-Induced Oxidative Stress In Vivo

**Figure 8:**
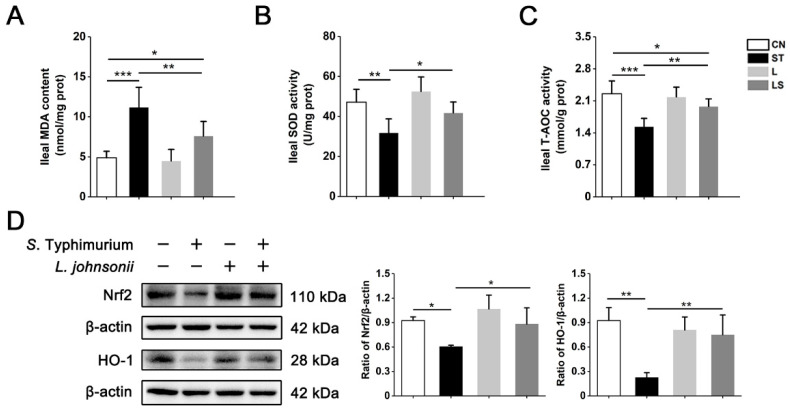
*L. johnsonii* L531 relieves *S*. Typhimurium-induced oxidative stress in vivo. The content of MDA (**A**), SOD (**B**), and T-AOC (**C**) in ileum (*n* = 6 mice, respectively). (**D**) Western blot analysis of Nrf2 and HO-1 in ileum. Values are expressed as the means ± SEM of 3 independent experiments. * *p* < 0.05, ** *p* < 0.01, *** *p* < 0.001.

To further investigate whether *L. johnsonii* L531 alleviates *S*. Typhimurium-induced oxidative stress in vivo, we examined SOD, T-AOC activity, and MDA content in ileum and liver. The results showed that *S*. Typhimurium caused a significant decreased activity of SOD and T-AOC and an obvious increase in MDA content in the ileum and liver (Figure 8A–C and Figure S2A–C). Interestingly, *L. johnsonii* L531 pretreatment relieved the oxidative damage, although in ileal T-AOC, MDA, and SOD, MDA indices of liver did not return to the same state as the control group; *L. johnsonii* L531 still efficiently reversed the oxidative damage compared with the *S*. Typhimurium group. Western blot analysis further confirmed that *L. johnsonii* L531 effectively reversed the inhibition of Nrf2 and HO-1 protein levels induced by *S*. Typhimurium in the ileum and liver (Figure 8D and Figure S2D). Collectively, these data implicate that *L. johnsonii* L531 alleviates oxidative stress caused by *S*. Typhimurium in vivo.

## 4. Discussion

**Figure 9:**
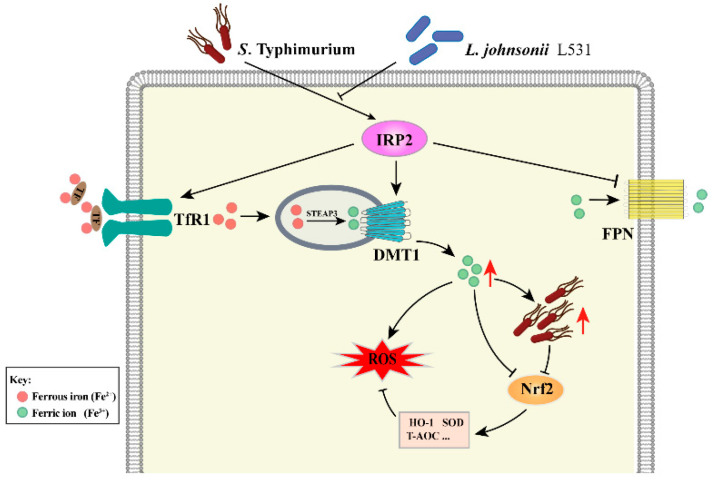
The main mechanism diagram of this study. *L. johnsonii* L531 reverses *S*. Typhimurium-induced iron metabolism disorder and oxidative stress via IRP2 pathway, thereby contributing to alleviating *S*. Typhimurium diarrhea.

Iron is an integral trace element fundamental to the life activities of all living things [24]. In this study, we report that *L. johnsonii* L531 effectively reverses *S*. Typhimurium-induced iron metabolism disorder and oxidative stress via the IRP2 pathway, thereby contributing to alleviating *S*. Typhimurium diarrhea (Figure 9).

Current studies have proven that *Salmonella* retains iron in cells and uses it to promote its own reproduction [6,25,26]. Consistently, we observed that *S*. Typhimurium load and iron content were significantly higher in cells, feces, and liver in this work. Furthermore, in line with the previous findings that *Lactobacillus* alleviates iron overload in tissue, and even is effective in relieving *S*. Typhimurium-induced intestinal damage in a high iron diet model [9,27], our study shows that *L. johnsonii* L531 pretreatment was beneficial in reversing serum iron deficiency and tissue iron overload. These data clearly indicate that *L. johnsonii* L531 has a crucial role in ameliorating iron metabolism disorder, highlighting the necessity to gain a deeper understanding of mechanisms of *S*. Typhimurium-induced iron metabolism disorder and the consequent protective effect exerted by *L. johnsonii* L531.

A variety of cells, including epithelial cells, can acquire iron via TfR1 and DMT [28,29] and maintain intracellular iron homeostasis by transporting iron to the outside of the cell via FPN [30]. *S*. Typhimurium prevents iron from being transported out of the cell by inhibiting FPN, resulting in the imbalance of iron homeostasis [26,31,32]. Indeed, we found that iron transporter protein FPN was markedly inhibited both in vitro and in vivo, while *L. johnsonii* L531 significantly reversed the inhibition of FPN.

Moreover, here, we also observed that *S*. Typhimurium infection induced an increase of DMT1 and TfR1. However, *L. johnsonii* L531 efficiently reversed the activation of DMT1 and TfR1 caused by *S*. Typhimurium. Thus, it is possible that *L. johnsonii* L531 regulates expression of the key iron transport proteins (DMT1, TfR1, and FPN) to attenuate iron overload, thereby maintaining balance of iron homeostasis. Concurrently, it is worth noting that recent studies have shown a non-significant change in the expression of DMT1 and TfR1 during *S*. Typhimurium infection in RAW264.7 cells [33]. The expression of TfR1 could even be significantly suppressed following *S*. Typhimurium infection and that DMT1 deficiency could exacerbate *S*. Typhimurium infection in BMDMs (bone marrow-derived macrophages) [32]. The regulation of host iron transport proteins is a dynamic process, and the level of protein expression depends on the mode of *Salmonella* infection and the time point of investigation, and expression pattern is various even in different cells [34]. Our results suggest that activation of DMT1 and TfR1 is involved in the regulation of iron homeostasis by *S*. Typhimurium in both IPEC-J2 cells and Hela cells, which is beneficial for the survival of *S*. Typhimurium.

In addition, IRP2 controls the transcripts of FPN, DMT1, and TfR1 as a central iron regulatory protein [14]. Notably, our data showed that *S*. Typhimurium caused high expression of IRP2, and knockdown of IRP2 effectively reduced the *S*. Typhimurium burden and reversed the inhibition of FPN and activation of DMT1 and TfR1 induced by *S*. Typhimurium. These results reveal a crucial role that IRP2 plays in the iron metabolism disorder caused by *S*. Typhimurium. It is possible that *S*. Typhimurium increased the expression of DMT1 and TfR1 by promoting the expression of IRP2. Interestingly, *L. johnsonii* L531 pretreatment could inhibit the increase in IRP2, TfR1, and DMT1, and reverse the decrease in FPN caused by *S*. Typhimurium, but not in Hela cells overexpressing IRP2. Together with these data, our findings further elucidate that *L. johnsonii* L531 against *S*. Typhimurium induced disorders of iron metabolism via the IRP2 pathway.

Iron overload has been identified as one of the main triggers of oxidative stress [16,35,36]. Additionally, recent studies have shown that *Salmonella* can cause high ROS in the host [3,37,38]. Our data found that *S*. Typhimurium caused the accumulation of ROS and highlighted the high ROS and inhibition on antioxidant-related factors caused by *S*. Typhimurium which is due to an imbalance in iron homeostasis. Further, we also observed that overexpression of IRP2 further aggravated cellular free divalent iron accumulation and oxidative stress induced by *S*. Typhimurium. IRP2 overexpression was also able to promote the expression of TfR1 and DMT1 and inhibit the expression of FPN. These results suggest that *S*. Typhimurium may upregulate the expression of DMT1 and TfR1 and suppress the expression of FPN by activating IRP2, thereby causing cellular iron overload and ultimately triggering oxidative stress.

*Lactobacillus* have been shown to mitigate oxidative stress through promoting the release of Nrf2 and its downstream antioxidant factors, such as HO-1, GPX1, and SOD, indicating beneficial antioxidant activity [36,39,40,41,42]. Consistently, *L. johnsonii* L531 pretreatment ameliorated the accumulation of ROS induced by *S*. Typhimurium with the activation of Nrf2 and its downstream antioxidant factors HO-1, SOD1, GPX1, and T-AOC. However, *L. johnsonii* L531 has little or no protective effect any more in protecting cells from the iron metabolism disorder and oxidative stress induced by *S*. Typhimurium following IRP2 overexpression. Moreover, knockdown IRP2 significantly reduced cellular free divalent iron content, expression of DMT1 and TfR1, and inhibited expression of FPN, while IRP2 overexpression showed the opposite trend compared to knockdown IRP2, and IRP2 overexpression caused oxidative stress to some extent. It is possible that the role of IRP2 protein as a special bridge between iron metabolism and oxidative stress, and more importantly, our results, reveal the beneficial role of *L. johnsonii* L531 in reversing the increased DMT1 and TfR1 and decreased FPN by blocking the activation of IRP2 caused by *S*. Typhimurium, which thereby attenuates iron overload and oxidative stress.

Intestinal oxidative stress adversely affects the digestion and absorption of nutrients, resulting in various intestinal disorders; iron overload could also induce intestinal damage and increase the risk of *Salmonella* infection [9,31]. Indeed, *S*. Typhimurium caused damage to the liver and ileum and the infected mice developed obvious diarrhea in our study. However, *L. johnsonii* L531 pretreatment remarkably reversed these pathological phenomena. Thus, it may be that iron overload further led to oxidative damage on intestine, ultimately causing diarrhea. It is also possible that *L. johnsonii* L531 relieves intestinal oxidative stress by maintaining the balance of host iron homeostasis, thereby alleviating intestinal injury and diarrhea. Moreover, recent studies have shown that metabolites of gut microbial, including *L. johnsonii* and *L. reuteri*, play crucial roles in modulating host iron homeostasis [27]. In this study, it is important to note that host iron homeostasis levels in the *L. johnsonii* L531 groups were maintained in relative equilibrium, including iron content and expression levels of IRP2, DMT1, TfR1, and FPN. Therefore, the metabolites of *L. johnsonii* L531 may have a key effect on enhancing the defensive capacity of cellular nutritional immunity against *S*. Typhimurium infection. Future work will determine the types and functions of metabolites produced by *L. johnsonii* L531.

## 5. Conclusions

The present findings confirm that *L. johnsonii* L531 efficiently reverses the increase in TfR1 and DMT1 and decrease in FPN induced by *S*. Typhimurium, thereby relieving iron overload and oxidative damage via the IRP2 pathway. These effects of *L. johnsonii* L531 contribute to ameliorating diarrhea and intestinal damage caused by *S*. Typhimurium. In addition, further research should focus on exploring the metabolites of *L. johnsonii* L531 involved in the regulation of iron metabolism, which will offer us opportunities to help drive targeted prevention and control of *S*. Typhimurium infection at a later stage. The prevention and control of animal-derived pathogenic bacteria is extremely important for animal-derived food safety in human life. Our observations represent a promising strategy for the potential application of *L. johnsonii* L531 in the defense against *S*. Typhimurium infection.

## Figures and Tables

**Table 1:** Information of oligonucleotide primers used for real-time qPCR and IRP2 eukaryotic expression vector construction.

Gene Primer	Direction	Sequence (5′-3′)	GenBank Accession
IRP1	F	CCGTGCCAATTATCTCGCCTCTC	XM_021078286.1
	R	TGCCCGTAGAGTCAGTACCTAAAGG	
SOD1 (Sus scrofa)	F	CTCTCGGGAGACCATTCCATCATTG	NM_001190422.1
	R	TTCTTCATTTCCACCTCTGCCCAAG	
GPX1 (Sus scrofa)	F	CACGCTCGGTGTATGCCTTCTC	NM_214201.1
	R	GCAGCTCATTCATCTGGGTGTAGT	
NQO1 (Sus scrofa)	F	CGTACAGCATTGGGCACACTCC	NM_001159613.1
	R	CAAAGTACAGTGGCGTCTCATCCC	
SOD1 (Homo sapiens)	F	GATGACTTGGGCAAAGGTGGAAATG	NM_000454.5
	R	CCAATTACACCACAAGCCAAACGAC	
GPX1 (Homo sapiens)	F	GCAACCAGTTTGGGCATCAGGAG	NM_000581.4
	R	CACCGTTCACCTCGCACTTCTC	
NQO1 (Homo sapiens)	F	AAGCCGCAGACCTTGTGATATTCC	NM_000903.3
	R	CATGGCAGCGTAAGTGTAAGCAAAC	
GAPDH (Sus scrofa)	F	GCTGCTGAACGGGAAGACAA	NM_001206359.1
	R	AGCACCAGCATCACCCCATTTG	
GAPDH (Homo sapiens)	F	GGAGCGAGATCCCTCCAAAAT	NM_001289746.2
	R	GGCTGTTGTCATACTTCTCATGG	
IRP2-xhol	F	CCGCTCGAGATGGACGCCCCAAGTGCA	
IRP2-BamHI	R	CGCGGATCCCTATGAGAATTTTCGTGCCACAAAGT	

F = forward; R = reverse.

## Data Availability

Not applicable.

## Supplementary Materials

The following supporting information can be downloaded at: https://www.mdpi.com/article/10.3390/nu15051127/s1, Figure S1: Western blot analysis of IRP2, DMT1, TfR1 and FPN in liver. Figure S2: *L. johnsonii* L531 relieves *S*. Typhimurium-induced oxidative stress in vivo.
