# Supplementary material for: Lactobacillus johnsonii L531 Ameliorates Salmonella enterica Serovar Typhimurium Diarrhea by Modulating Iron Homeostasis and Oxidative Stress via the IRP2 Pathway
Source: Nutrients. 2023 Feb 23;15(5):1127. doi: 10.3390/nu15051127 (PMC10005772; doi:10.3390/nu15051127)
Supplement: Supplementary file 1 [file nutrients-15-01127-s001.zip › nutrients-2127024-Supplemental Materials.pdf]

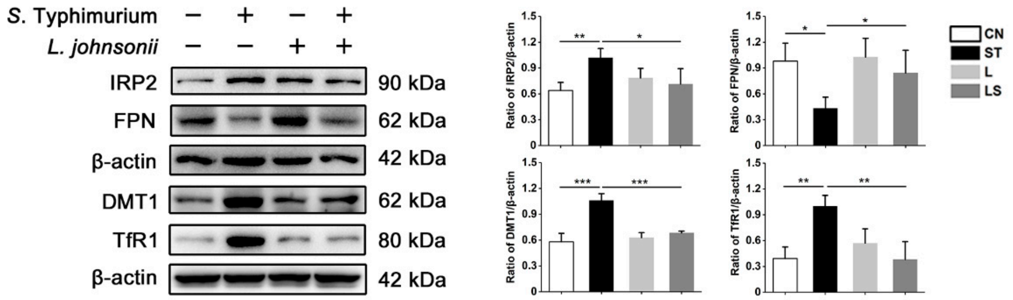

Figure S1. Western blot analysis of IRP2, DMT1, TfR1 and FPN in liver. *$P < 0.05$, **$P < 0.01$, ***$P < 0.001$.

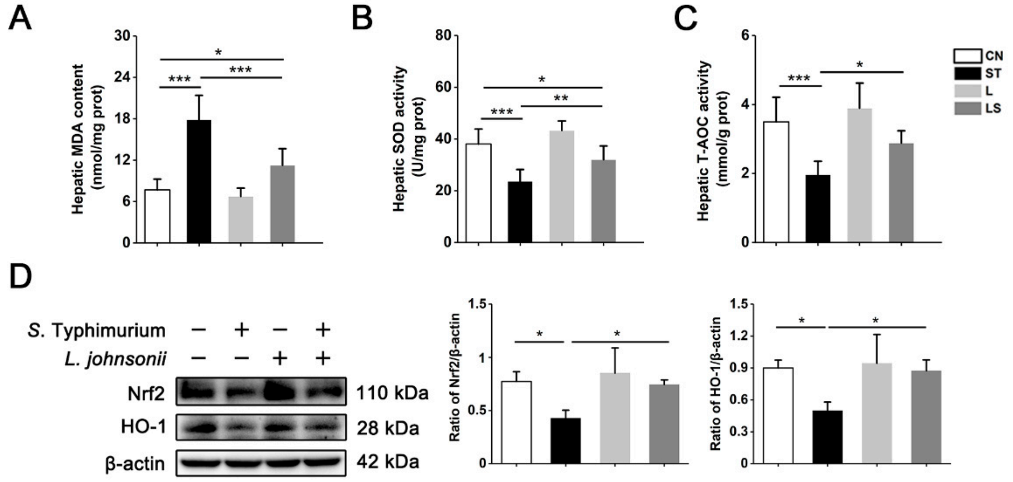

Figure S2. *L. johnsonii* L531 relieves *S*. Typhimurium-induced oxidative stress *in vivo*. The content of MDA (**A**), SOD (**B**), T-AOC (**C**) in liver (n = 6 mice, respectively). (**D**) Western blot analysis of Nrf2 and HO-1 in liver. *$P < 0.05$, **$P < 0.01$, ***$P < 0.001$.
